# Decolorization and biodegradation of melanoidin contained in beet molasses by an anamorphic strain of *Bjerkandera adusta* CCBAS930 and its mutants

**DOI:** 10.1007/s11274-020-02944-w

**Published:** 2020-12-22

**Authors:** Teresa Korniłłowicz-Kowalska, Kamila Rybczyńska-Tkaczyk

**Affiliations:** Department of Environmental Microbiology, Laboratory of Mycology, The University of Life Sciences, Leszczyńskiego Street 7, 20-069 Lublin, Poland

**Keywords:** Molasses, Decolorization, Detoxification, Immobilization, Peroxidase, *B. adusta* mutants

## Abstract

We used a ligninolytic strain of the white-rot fungus *B. adusta* CCBAS 930 and its mutants with modified ligninolytic activity to assess their potential to remove of molasses. The analyzed strains have been shown to be able to decolorize 1% or 2% molasses solutions containing brown-colored toxic melanoidins. It was found that the decolorization process was determined by the transition to the stage of production of sporulating aerial mycelium (liquid and agar cultures) coupled with an increase in peroxidase activity, which was accompanied by a decrease in the level of melanoidin, free radicals, and phenolic compounds. Four different peroxidase activities were detected in post-culture liquids, i.e. horseradish-like (HRP-like), manganese-dependent (MnP), lignin (LiP), and versatile (VP) peroxidase activities. The HRP-like peroxidase was characterized by the highest activity. The efficiency of removal of melanoidins from a 1% molasses solution by the parental strain and the mutants was dependent on the culture method. The highest efficiency was noted in immobilized cultures (threefold higher than in the mycelium-free cultures), which was accompanied by stimulation of HRP-like peroxidase activity. Mutant 930-5 was found to be the most effective in the decolorization and decomposition of melanoidin. The HRP-like activity in the immobilized cultures of *B. adusta* 930-5 was 640-fold higher than in the mycelium-free cultures of the fungus. Moreover, decolorization and biodegradation of melanoidin by *B. adusta* CCBAS 930 and 930-5 was coupled with detoxification.

**Electronic supplementary material:**

The online version of this article (10.1007/s11274-020-02944-w) contains supplementary material, which is available to authorized users.

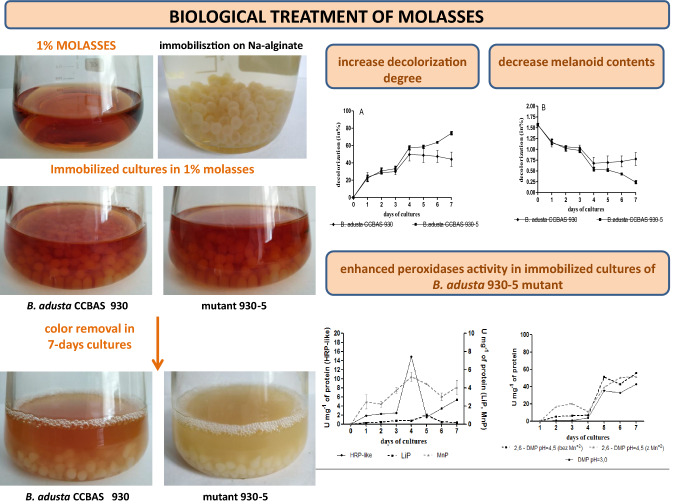

## Introduction

Beet molasses is a sugar industry by-product containing from 48 to 50% of sugars (sucrose, glucose, fructose, raffinose) nitrogen compounds, organic acid, amino acids etc. (Miranda and Benito 1996; Kotzamanidis et al. 2002). Besides Poland, beet molasses is mostly produced in warm climates and in the USA, Germany, France, and Turkey (Akar and Canbaz 2016). Li et al. (2015) reported a total sugar worldwide yield of 0.16 billion tons in 2011. Approximately 0.3 tons of molasses is discarded while 1 ton of sugar is processed (Li et al. 2015). The annual sugar production in Poland is estimated at 1700 thousand tons (Kowalczyk-Juśko et al. 2014), which is associated with production of 510 thousand tons of molasses. As reported by Guc and Erkurer (2017), the annual production of beet molasses in Turkey in 2014 exceeded 710 thousand tons. The main application of molasses is the production of bioethanol (Razmovski and Vučurović 2012). In Polish distilleries, approximately 15% of beet molasses are used for production of ethanol (Grajek et al. 2008). Another application of molasses in the fermentation industry is the production of organic acids, i.e. oxalic, citric, lactic, and acetic acids (Gur et al. 2001; Kotzamanidis et al. 2002; Guc and Erkmen 2017), and amino acids (Kahraman and Yesilada 2003). Molasses is also a raw material for production of feed and baker's yeast in the yeast industry (Kahraman and Yesilada 2003; Li et al. 2015).

A serious problem in the industrial use of molasses as a carbon source for microorganisms producing alcohol and other bioproducts is the presence of colored toxic compounds called melanoidins. They are natural substances generated in the non-enzymatic Maillard amino-carbonyl reaction (Fitzgibbon et al. 1995). These hardly degradable organic compounds are obtained from heated mixtures containing reducing sugars with amino acid or proteins in the sugar production process (Arimi and Zhang 2014). Melanoidins are also present in molasses spent wash (MSW) and vinasse, which are wastes from ethanol production and other fermentation processes (Pant and Adholeya 2007). For example, 3.25·10^9^ l of alcohol are produced and simultaneously 40.4·10^10^ l of the waste are generated by 319 distilleries in India (Pant and Adholeya 2007). In terms of chemistry, melanoids resemble humic compounds. Yet, their chemical structure is still unclear (Seyis and Subasioglu 2009; Fan and Nguyen 2011). Melanoidins are acidic polymeric dispersed colloids with carboxylic and phenolic groups (Migo et al. 1993). They are highly dangerous to the environment. They exhibit antioxidant properties, toxicity against many microorganisms, and a harmful effect on the biological life in waters, since the dark color of wastewater containing these compounds blocks the access to light, thereby inhibiting photosynthesis and inducing anaerobic conditions (Fitzgibbon et al. 1995; Fahy et al. 1997; Singh et al. 2007; Raghukumar and Rivonkar 2001). Molasses spent wash (MSW) is highly hazardous to the environment due to its high acidity (pH from 4.0 to 4.3) and high chemical oxygen demand (COD) in the range of 25–30,000 mg/L (Pant and Adholeya 2007; Singh et al. 2007); hence, it may contribute to eutrophization of rivers or other water bodies (Kahraman and Yesilada 2003). Additionally, melanoidins reduce soil fertility by causing manganese deficiency, as they inhibit the conversion of Mn (II) to Mn (IV) (Chopra et al. 2004), reduce soil pH, and inhibit seed germination (Fitzgibbon et al. 1995). Given the threat to the environment, the key but still unresolved issue is the decolorization of molasses wastewater. Removal of the color with conventional methods is difficult and criticized (Pant and Adholeya 2007). There is a need for new methods for MSW and vinasse decolorization based on specific microorganisms. Therefore, biological decolorization of molasses melanoids seems to be the most appropriate mode of removal of these toxic compounds due to the environmentally friendly character and low costs of the method. Studies on decolorization of molasses and molasses spent wash conducted to date have demonstrated that some microorganisms, including bacteria and fungi, have an ability to remove color (Sirianuntapiboon et al. 1988; Sirianuntapiboon and Zohsalam 2004; Nakajima-Kambe et al. 1999; Jiménez et al. 2003; Seyis and Subosioglu 2009; Murata et al. 1991; Raghukumar and Rivonkar 2001; Chen et al. 2016). Bioremediation of melanoidin-containing wastewater by white-rot fungi and their ligninolytic enzymes has been reported as well (Thakker et. al. 2006; Fahy et al.^1997^; Kim and Shoda 1999; Singh et al. 2007; Kahraman and Yesilada 2003). Fungi used for bio-treatment of molasses wastewater grow rapidly due to the presence of carbon sources, which is correlated with the ability to decompose organic pollutants by extracellular ligninolytic and hydrolytic enzymes (Satyawali and Balakrishnan 2008). White-rot fungi *Phanerochaete chrysosporium* and *Trametes (Coriolus) versicolor* have been investigated most widely to assess their ability to decolorize melanoids (Dahiya et al. 2001; Benito et al. 1997; Chopra et al. 2004; Fahy et al. 1997). Their molasses decolorizing activity was coupled with the production of intracellular peroxidases (Maza et al. 2015; Satyawali and Balakrishnan 2008), e.g. Mn-dependent (MnP) and lignin (LiP) peroxidases. Maza et al. (2015) demonstrated high activity of laccase in *Trametes* sp. and *Phanerochaete* sp. cultures in molasses-amended media. However, little is known to date about the decolorization of melanoids by fungi from the genus *Bjerkandera* sp. (Maza et al. 2015).

Studies on fungal metabolic activity are primarily carried out in liquid cultures, in which fungi form “felt” on the surface of the medium (stationary cultures) or agglomerates inside the substrate (shaken cultures). A different type is liquid culture with biological material (hyphae) immobilized on the surface or inside the carrier. The use of such immobilized cultures for biodegradation of aromatic compounds has many advantages. Immobilized cells are more stable, durable, and resistant to environmental conditions than conventional cultures (Bouabidi et al. 2018; Kurade et al. 2019).

We used a ligninolytic strain of the white-rot fungus *B. adusta* CCBAS 930 and its mutants with modified ligninolytic activity to assess their potential to remove molasses. It was initially assumed that the removal of melanoidins from solutions by the *B. adusta* CCBAS 930 strain proceed via an enzymatic pathway. This thesis was confirmed by determination of the activity of ligninolytic enzymes (peroxidase and laccase) level of melanoids, free radicals, and phenolic compounds as products of biotransformation of molasses. Phyto- and biotoxicity assays were carried out to examine whether the process of decolorization and biodegradation of melanoids was accompanied by detoxification thereof. The investigations also consisted in immobilization of the mycelium (on Na-alginate beads) to increase the efficiency of removal of melanoidins from the solution.

## Material and methods

### Fungal strain

The soil anamorphic (conidial) basidiomycete *Bjerkandera adusta* CCBAS 930 strain and its four phenotypic mutants 930-2, 930-5, 930-14, and 930-20 were used in the study. The isolation and identification based on morphological traits and ITS1, 5,8S rRNA, and ITS2 gene sequences of the parental *B. adusta* CCBAS 930 strain are presented in the paper by Korniłłowicz-Kowalska et al. (2006a) and Korniłłowicz-Kowalska and Rybczyńska (2012). The identification sequences of the wild strain of the fungus are deposited in the GenBank database under accession number AY319191. The culture is deposited as *B. adusta* CCBAS 930 in the Culture Collection of Basidiomycetes, Prague, the Czech Republic. The induction and selection of *B. adusta* CCBAS 930 mutants with ligninolytic activity modified by the application of N-methyl N-nitro-N-nitrosoguanidine (NTG) and UV irradiation is described in the study conducted by Korniłłowicz-Kowalska and Iglik (2007) as well as Korniłłowicz-Kowalska and Rybczyńska (2010). The UV irradiation (UV-C 200–280 nm) and nitrosoguanidine treatment of the mycelium homogenate of the parental *B. adusta* CCBAS 930 strain yielded 7 strains with altered colony morphology (felting and yellowish discoloration of the mycelium) and more effective (except for one strain) decolorization of 0.2% post-industrial lignin solutions in comparison with the parental strain (Korniłłowicz-Kowalska and Rybczyńska 2010). The genotypic differences between the parental *B. adusta* CCBAS 930 strain and its two phenotypic mutants 930-5 and 930-14 were confirmed by RAPD-PCR analysis carried out with the use of OPN primers for molecular typing, as presented by Korniłłowicz-Kowalska and Rybczyńska (2014). The other two strains, i.e. 930-2 and 930-20, with modified growth characteristics were not subjected to the preliminary RAPD-PCR analysis due to the poor mycelial growth. The mutants selected for the present investigations, i.e. 930-2, 930-5, 930-14, and 930-20, were previously denoted as R59-2, R59-5, R59-14, and R59-20, respectively, whereas the parental CCBAS 930 strain was assigned as R59 (Korniłłowicz-Kowalska and Iglik 2007). The parental strain and the fungal mutants were kept on PDA slants at + 4 °C.

### Molasses

Molasses, i.e. a by-product of beet sugar production (sucrose), was obtained from the Lublin sugar factory (Poland) and kept at + 4 °C. Its chemical characteristics are shown in Table 1S. The following determinations were carried out: total N content with the Kjeldahl method, protein level with the modified Kjeldahl method (Kjel-Tec analyzer), reducing sugars with high performance liquid chromatography (HPLC), mineral components (Na, K, Ca, Mg, Zn, Cu, Fe, Mn) with the flame AAS method, and quantitative and qualitative composition of amino acids with the HPLC method. The content of melanoidins in the molasses was determined spectrophotometrically (A400nm) as in Migo et al. (1993) based on a standard curve prepared for synthetic melanoidins. To this end, 1L of a solution containing 1 M glucose, 1 M glycine, and 0.5 M Na_2_CO_3_ in distilled water was prepared and autoclaved at 121 °C for 3 h. The stock solution of synthetic melanoidins was a basis for making dilutions for the standard curve.

### Cultures of *B. adusta* CCBAS and its mutants

Preliminary analyses of the abilities of the parental strain and its mutants to decolorize molasses (M) were carried out on solid Park and Robinson (1969) medium without glucose addition (g/L): NH_4_NO_3_ –0.1; KH_2_PO_4_ –0.2; MgSO_4_ × 7H_2_O–0.5; agar 20 g, H_2_O–1L, with 1% and 2% molasses supplementation, using a mycelium disc with ø = 1 cm from a 7-day culture on PDA medium as an inoculum. The other experiments with molasses and mycelium-free cultures were carried out in stationary cultures on liquid Park and Robinson medium supplemented with 1% molasses without addition of glucose. All liquid cultures were carried out in 200 mL Erlenmayer flasks containing 100 mL of medium (molasses were added after sterilization of the medium). 1 mL of mycelium homogenate containing 10^6^ c.f.u./mL obtained through homogenization of a 7-day culture on liquid glucose-potato (PD) medium was used as the inoculum. The density of the inoculum was established by plating dilutions of homogenized mycelium suspensions. The cultures were incubated at 26 °C for 3 weeks.

### Preliminary study of crude post-culture liquids of the *B. adusta* CCBAS 930 strain and its four mutants

Transparent post-culture liquids obtained by filtration of the mycelium followed by centrifugation of cultures of the parental fungal strain (*B. adusta* CCBAS 930) and all mutants (930-2, 930-5, 930-14, and 930-20) were subjected to periodic determinations (after 0, 3, 7, 10, 14, 18, and 21 days) of the molasses decolorization rate at A400nm (maximum absorbance), concentration of phenols (A400 nm) according to Malarczyk (1984) using the analytical curve for protocatechuic acid (µg/mL), protein content with the Lowry method (Lowry et al. 1954) using bovine albumin as a protein standard, content of reducing sugar with the Samogyi-Nelson method using a glucose calibration curve and pH of post-culture liquids with the potentiometric method.. The activities of horseradish-type peroxidase (HRP-like) were estimated according to Maehly and Chance (1954) with slight modification (Rybczyńska-Tkaczyk et al. 2020) using 255 µL of 0.01% o-dianisidine (ε_460nm_ = 11.3 M^−1^ cm^−1^). Laccase activity was estimated according Leonowicz and Grzywnowicz (Leonowicz and Grzywnowicz 1981) using syringaldazine as a substrate.

A broader spectrum of activity of extracellular oxidoreductases, including HRP-like peroxidases, laccase and manganese-dependent (MnP), lignin (LiP), and versatile (VP) peroxidases, was studied in the stationary cultures of the wild *B. adusta* CCBAS 930 strain and its mutant 930-5, which exhibited the highest efficiency of decolorization of 1% molasses solutions of all the investigated mutants. The activities of extracellular oxidoreductases were estimated according to an available assay with own modification (Rybczyńska-Tkaczyk et al. 2020). The activity of manganese-dependent peroxidase (MnP) (Wariishi et al. 1992) was determined by oxidation of 15 µL of 1 mM MnSO_4_ in 265 µL of sodium malonate (50 mM, pH 4.5) in the presence of 50 µL of supernatant and 10 µL of 6 mM H_2_O_2_, and subsequent determination of the Mn^+3^–malonic acid complex (ε_270nm_ = 11,590 M/cm). The activity of lignin peroxidase (LiP) was assayed using 20 mM veratryl alcohol (ε_310nm_ = 9300 M/cm) (Tien and Kirk, 1988) in 40 mM tartrate buffer, pH 3, in the presence of 10 µL of 8 mM H_2_O_2_. Versatile peroxidase (VP) activity was assessed by oxidation of 2,6-dimethoxyphenols (2,6-DMP). The Mn-independent activity of VP was estimated by oxidation of 15 µL of 20 mM 2,6-DMP (*ɛ*_468_ = 49.6 M/cm) in 265 µL of 50 mM sodium malonate buffer (pH 3.0 and 4.5) with 10 µL of 6 mM H_2_O_2_. The Mn-dependent activity of VP was determined by oxidation of 15 µL of 20 mM 2,6-DMP in 250 µL of 50 mM sodium malonate buffer (pH 4.5) in the presence of 10 µL of 6 mM H_2_O_2_ and 15 µL of 0.1 mM MnSO_4._

### Enhancement of molasses removal using immobilized mycelium of *B adusta* CCBAS 930 and its mutant 930-5

100 mL of a sterile Na-alginate solution (4%) were mixed with 20 g of homogenized mycelia of *B. adusta* CCBAS 930 and its mutants 930-5 and agitated (150 rpm, 20 min). The mixture (25 mL) was extruded using a sterile syringe into 0.2 M CaCl_2_, thus forming beads with a 3.0–4.0 mm diameter. The beads were allowed to harden in 0.2 M CaCl_2_ for about 24 h at 4 °C. Next, the 0.2 M CaCl_2_ solution was removed and the beads were washed twice with the addition of distilled water. Immobilized mycelium (Na-alginate beads) of *B. adusta* CCBAS 930 was incubated with 1% solutions of molasses (150 rpm, 28 °C, 7 days). Samples were collected every day and the following parameters were estimated: the degree of decolorization of molasses (A400nm), the concentrations of phenolic compounds and the activity of peroxidases: horseradish-type (HRP-like), ligninase (LiP), manganese-dependent (MnP), versatile (VP) and laccase (Lac) (2.4). The relative level of free radicals was estimated spectrophotometrically based on the detection of superoxide-induced formation of formazan from nitrotetrazolium blue (NBT). The reaction mixture was prepared with 3 mL of distilled water, 0.05 mL of 1 M NaOH, 0.1 mL of a 5 mM NBT solution, and 0.2 mL of the sample and incubated (30 min at 20 °C). The absorbance was measured at 560 nm (Paździoch-Czochra et al. 2003).

### Morphological observations of stationary cultures of *B. adusta* CCBAS 930 and its mutants

Throughout the experiment, macroscopic observations of the morphology of aerial and substrate mycelia and changes in the medium color were carried out.

### Resazurin reduction bacterial viability assay

The molasses and synthetic melanoidin solutions and post-culture fluids from the mycelium-free and immobilized cultures of the *B. adusta* CCBAS 930 strain and its 930-5 mutant were tested against Gram-positive *Staphylococcus aureus* ATCC 29737 and Gram-negative *Eschericha coli* ATCC 25922. The strains were provided by ATCC and stored at 4ºC. All strains were cultured on nutrient broth (NB) medium at 37ºC. Resazurin is a non-toxic water-soluble dye previously applied in bacterial viability studies (Osaka and Hefty 2013). This assay is based on detection of the metabolic activity of the cells. The redox dye resazurin (7-hydroxy-3H-phenoxazin-3-one 10-oxide) enters the cell in the oxidized form (blue), where it is converted to a reduced form, resorufin (pink). The reduced and oxidized forms of resazurin can be measured separately with a spectrophotometer and used to determine the reduction capability of cells, which reflects the mitochondrial function and cell viability and shows time- and concentration-dependent cell growth inhibition. Serial twofold dilutions of molasses and synthetic melanoidins were made with Mueller Hinton Broth (MHB) to yield final concentrations ranging from 10 to 0.62 mg/mL (1–0.062%) and from 5 to 0.31 mg/mL (0.5–0.031%), respectively, and placed into a 96-well plate. The bacterial suspension (100 µL) prepared from an overnight culture was adjusted to inoculation of 10^8^ CFU/mL. Then, 100 μL of bacterial culture were added. The wells with MHB or yeast culture were the negative and positive control, respectively. The plates were incubated at 37 °C for 48 h. Then, 20 μL of a 60-μM resazurin solution in PBS buffer were added to each well. After incubation (2 h, 37 °C), the viability of cells was monitored by measuring absorbance at 570 nm (reduced) and 600 nm (oxidized) (Osaka and Hefty 2013) and calculating the resazurin reduction factor (RRF).

### Phytotoxicity assay

The phytotoxicity assay was performed for the untreated medium with 1% molasses and supernatants obtained after 21-day stationary cultures and 7-day immobilized cultures of *B. adusta* CCBAS 930 and its 930-5 mutant. The phytotoxicity assay was carried out to determine root growth inhibition (I) and germination capacity (GI) in *Lepidium sativum.* L*.* seeds before and after decolorization (Rybczyńska-Tkaczyk and Korniłłowicz-Kowalska, 2017). Petri dishes were lined with filter paper and, subsequently, 100 seeds and 5 mL of the filtered (Ø = 0.22 µm) sample were added. The dishes were incubated at room temperature for 72 h. Distilled water was used as a control.

### Data analysis

The results were presented as a mean of three repetitions for which standard deviation was calculated. Correlation coefficients between the decolorization degree and the phenolic levels and peroxidase activity in the liquid culture media were estimated.

## Results

### Decolorization of molasses by *B. adusta* CCBAS 930 and its mutants

**Fig. 1:**
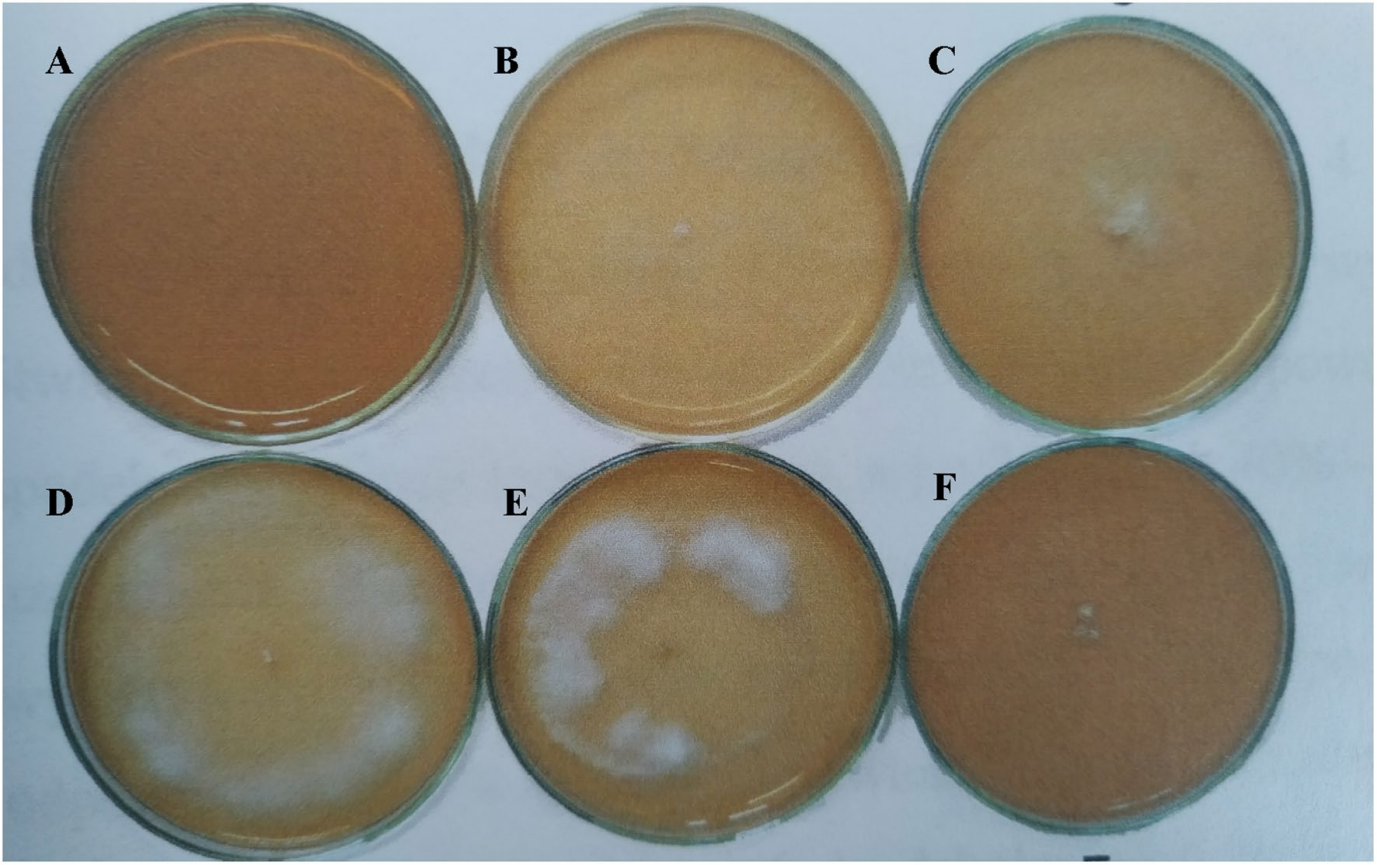
Decolorization of 1% molasses in 6-days cultures of *B. adusta* CCBAS 930 and its mutants (**a** control, **b** *B. adusta* CCBAS 930, **c** 930-2, **d** 930-5, **e** 930-14 and **f** 930-20) on agarized Park and Robinson medium

The parental strain and its four mutants exhibited a diverse ability to decolorize 1% molasses in the solid medium. The fastest decolorization of the medium, i.e. after 6 days, was visible in the cultures of the parental strain (930) and mutant 930-5. This was manifested in the brightening of the substrate over the entire surface of the plate (ø = 90 mm), which corresponded to the diameter of the colony and its aerial mycelium (Fig. 1; Table 2S). Mutant 930-14 turned out to be a slightly weaker molasses decolorizer: 55% of the medium was decolorized at a colony diameter equal to the diameter of the plate. The other two mutants only slightly (10–20%) brightened the agarized 1% molasses solution (Fig. 1; Table 2S). In solid cultures containing 2% molasses, only the parental strain brightened the medium over the entire surface of the plate (cultivation day 6), whereas the mutants did not decolorize the molasses in these conditions (Table 2S).

**Fig. 2:**
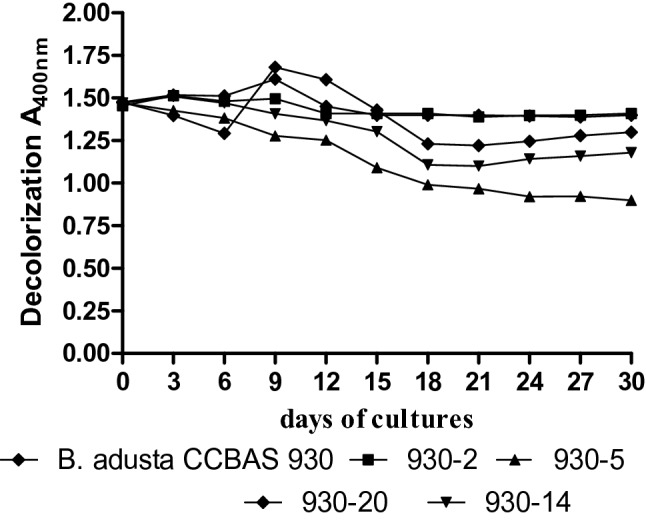
Decolorization (A_400nm_) of 1% molasses in post-cultures liquids parental strain of *B. adusta* CCBAS 930 and its mutants: 930-2, 930-5, 930-20 and 930-14

The use of the mycelium-free liquid medium slowed down the rate of molasses brightening, which was associated with slower production of aerial mycelium. In the first week, the strains grew inside the substrate and the growth of aerial mycelium coinciding with brightening of the molasses solution was noted only at the end of the incubation period. The most efficient decolorization of the 1% molasses solutions was achieved by mutant 930-5, followed by 930-14, i.e. 33% and 25% loss of color after 18 days of culture, respectively.

(Fig. 2). This effect was more pronounced in the case of mutant 930-5, which was characterized by production of more abundant aerial mycelium. A low level of decolorization of the 1% molasses solutions was demonstrated by the other mutants and the parental strain. The growth of the aerial mycelium in these strains was weaker as well (Table 3S). The use of the immobilized mycelium of the parental *B. adusta* CCBAS 930 strain and the most effective strain 930-5 significantly accelerated the process of decolorization of 1% molasses. After 7 days, the immobilized mycelium of both strains removed 44.16 and 74.32% of the compound, respectively. In the case of strain 930-5, an evident over 30% decrease in the intensity of the color of the medium was noted after 3 days (Fig. 2a, b).

### Changes in the melanoidin contents in fungal post-culture liquids

**Fig. 3:**
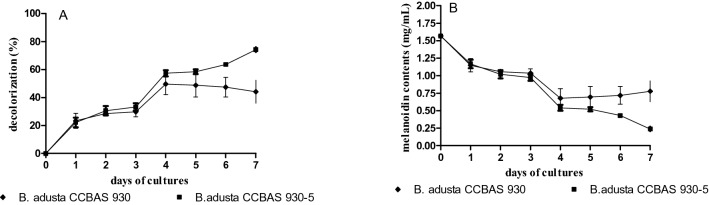
Decolorization (**a**) and melanoidin contents (**b**) during biotransformation of 1% molasses by immobilized mycelium of *B. adusta* CCBAS 930 and 930-5 mutant

Immobilization of the parental *B. adusta* CCBAS 930 and mutant 930-5 strains accelerated the removal of melanoidins. The most efficient decline in the melanoidin content was noted in the variant with the immobilized mycelium of mutant 930-5, where an 84.73% decrease was recorded in 7-day cultures (Fig. 3a, b).

### Changes in the concentration of phenols and free radicals (ROS) in fungal post-culture liquids

**Fig. 4:**
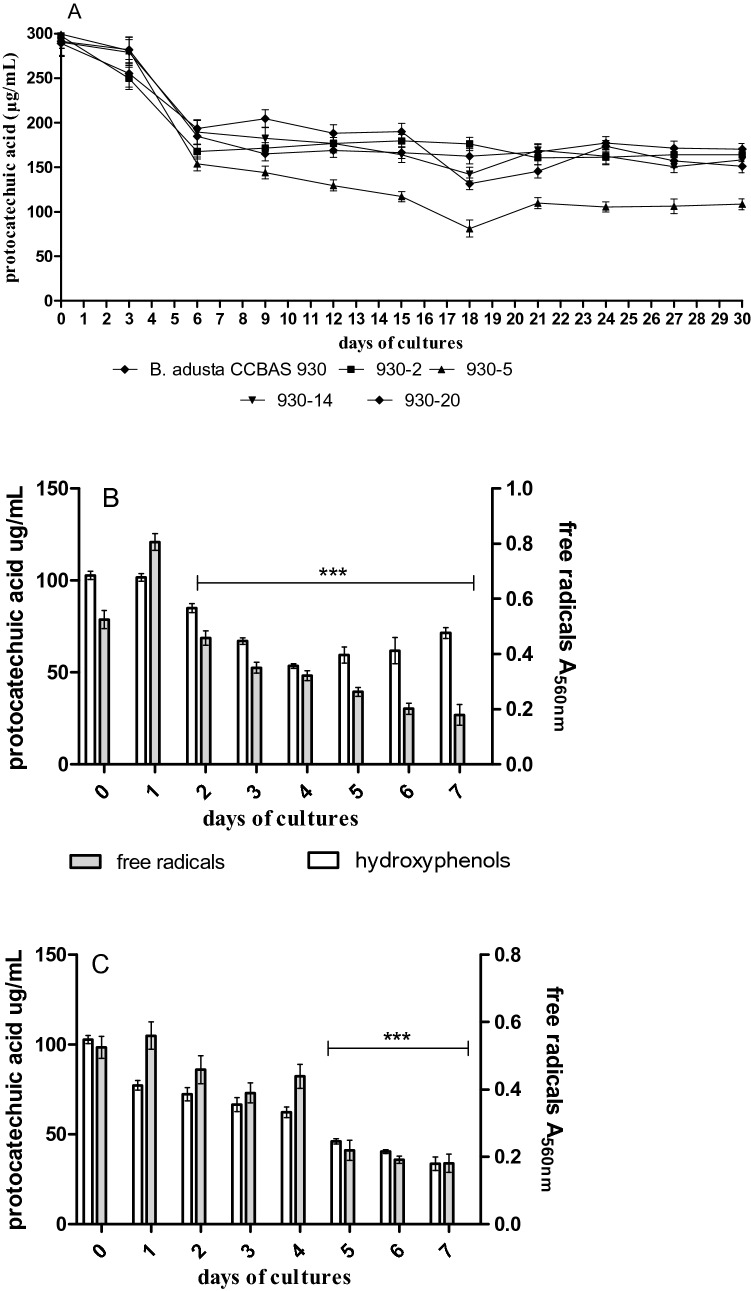
The content of phenols (µg/mL of protocatechuic acid) and level of free radicals (A_560nm_) in mycelium free *B. adusta* CCBAS 930 and its mutants (**a**) and in immobilized cultures of *B. adusta* CCBAS 930 (**b**) and immobilized cultures of its mutant: 930-5 (**c**)

As shown in Fig. 4a, phenolic compounds in the culture media of parental strain and its four mutants gradually declined. This process exhibited the highest rate in the first week of culture. After 6 days of the growth of the strains, the decrease in the phenol level ranged from 33% to approx. 49%. Phenols were most efficiently removed by strain 930-5, which reduced ¾ of their initial concentration after 18 days (73%). Over this time, 44% (930-20)—51% (930-14) reduction of the concentration of phenols was achieved by the other mutants and a 55% decrease was induced by the parental strain. A slight increase (10–20%) in the phenol level relative to the lowest content was observed after 18 days of cultivation (Fig. 4a). The content of phenolic compounds also decreased in the immobilized cultures of the parental strain and mutant 930-5; the change was most pronounced in the case of the mutant, which reduced their content by 25% after 24 h and by over 65% after 7 days (Fig. 4b, c).

Both in the stationary mycelium-free cultures and in the variant with immobilized mycelium of the parental *B. adusta* CCBAS 930 strain and mutant 930-5, there was an over 60–66% decrease in the level of free radicals. In the immobilized mycelium variant, the content of free radicals increased significantly after 24 h but systematically declined within the subsequent days (Fig. 4b, c).

### Utilization of sugar in *B. adusta* CCBAS 930 and mutant 930-5 cultures

**Fig. 5:**
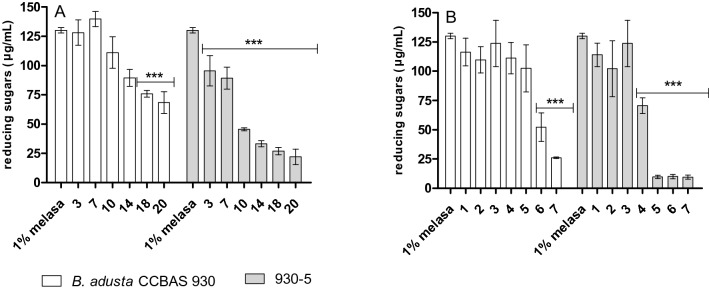
The content of reducing sugars in mycelium-free (**a**) and immobilized cultures (**b**) of *B. adusta* in CCBAS 930 and its 930-5 mutants supplemented with 1% molases (The significant difference was calculated using one-way ANOVA and post-Tukey test ***p < 0.001, versus the control 1% molasses medium

The sugar content was found to decrease in the mycelium-free culture and in the variant with the immobilized mycelium of the *B. adusta* CCBAS 930 strain and its mutant 930-5. A 47.45% decline in the content of reducing sugars was noted in the third week of the study in the mycelium-free *B. adusta* CCBAS 930 cultures. In the case of the mycelium-free cultures of mutant 930-5, a 26.60% decline in the content of reducing sugars was noted already in the first experimental week and 83.10% reduction was detected at the end of the culture. In the variant with immobilized mycelium of the parental *B. adusta* CCBAS 930 strain and its mutant 930-5, a significant decrease by 79.94% and 92.66%, respectively, was noted between days 5 and 7 (Fig. 5).

### Activity of extracellular fungal peroxidases

**Fig. 6:**
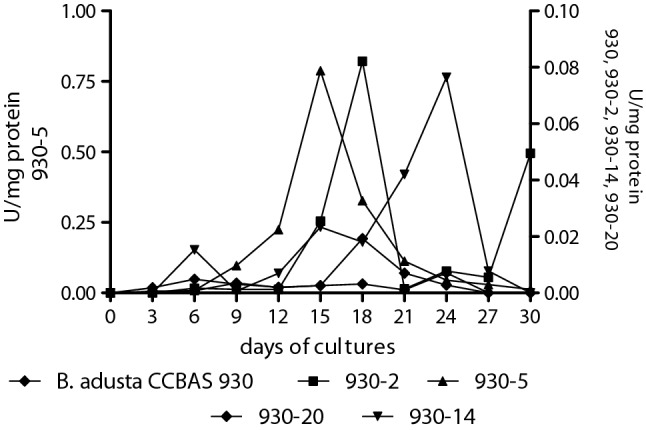
Activity of HRP-like peroxidase (U/mg) in culture of *B. adusta* 930 and its mutants with 1% molasses

It was found that the decolorization of the molasses-containing medium in the mycelium-free cultures was correlated with an increase in the activity of horseradish-like (HRP-like) peroxidase. The highest activity of this enzyme was exhibited by mutant 930-5 (Fig. 6). The maximum HRP-like peroxidase activity was noted on culture day 15 (0.7 U mg^−1^ protein). It preceded the maximum decolorization of molasses by this strain (day 18). At the time of full decolorization, the HRP-like peroxidase activity was already by 50% lower than its maximum. From culture day 21, the activity of this enzyme in the cultures of strain 930-5 was very low and disappeared after 30 days (Fig. 6). The peroxidase activity of the parental strain and the other three mutants was low throughout the study period. At its maximum (strains 930-2, 930-14), the activity was from 8 to 9 times lower (0.082 and 0.076 U/mg protein, respectively) than the maximum activity for strain 930-5 and 252 times lower than that of the parental strain (0.018 U/mg protein). The activity of this enzyme in the strain 930-20 cultures was in the range of 0.61–3.50 mU/mg protein throughout the study period.

**Fig. 7:**
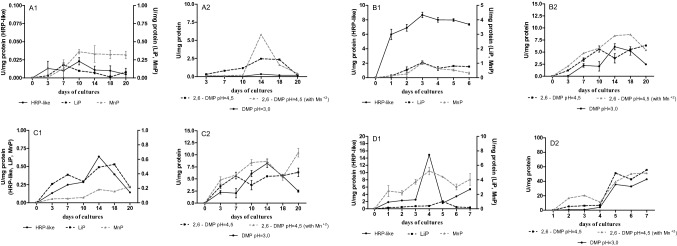
Activity of peroxidases (U/mg) in cultures of *B. adusta* CCBAS 930 (**a**1, **a**2—stationary cultures; **b**1, **b**2—immobilized cultures) and its 930-5 mutant (**c**1, **c**2—stationary cultures; **d**1, **d**2—immobilized cultures); the Mn-independent activity of VP was estimated by oxidation of 2,6-DMP in pH 3.0 and 4.5 and Mn-dependent activity of VP was determined by oxidation of 2,6-DMP in pH 4.5 in the presence of MnSO_4_

A broader spectrum of activity of peroxidases, including manganese-dependent (MnP), lignin (LiP), and versatile (VP) peroxidases, was studied in the stationary and immobilized cultures of the wild *B. adusta* CCBAS 930 strain and its mutant 930-5, which exhibited the highest efficiency of decolorization of 1% molasses solutions of all the investigated mutants (Fig. 7a and d1, d2). Laccase activity was not detected. The presence of all these oxidoreductases was detected in the cultures of both strains, with substantially higher activities in the immobilized cultures, especially in the case of mutant 930-5 (Fig. 7b and d1, d2). In the stationary mycelium-free cultures, the parental *B. adusta* CCBAS 930 strain was characterized by low levels of HRP-like, MnP, and LiP peroxidase activities not exceeding 0.005–0.098 U/mg protein. Higher activities in the stationary cultures of *B. adusta* CCBAS 930 were determined for versatile peroxidase VP (0.14–5.80 U/mg protein), whose maximum activity was detected on days 14–18 of the experiment. In the case of the immobilized cultures of the parental *B. adusta* CCBAS 930 strain, the activity of MnP and LiP peroxidases increased about tenfold and reached a maximum on day 3 (Fig. 7b1). In turn, a 340-fold increase was found in the case of the HRP-like activity (Fig. 7b1). VP was the only peroxidase whose activity increased insignificantly in the immobilized cultures of this strain, in comparison with the stationary culture conditions (Fig. 7a2, b2). The immobilized mycelium of mutant 930-5 was characterized by the highest activity of the tested peroxidases (Fig. 7d1, d2). The highest increase in the activity was observed for HRP-like peroxidase, i.e. an over 24-fold increase compared with the stationary cultures of this strain and a 640-fold increase in the activity compared with the parental *B. adusta* CCBAS 930 strain (Fig. 7d1).

### Bio- and phytotoxicity of *B. adusta* CCBAS 930 and mutant 930-5 post-culture liquids

**Fig. 8:**
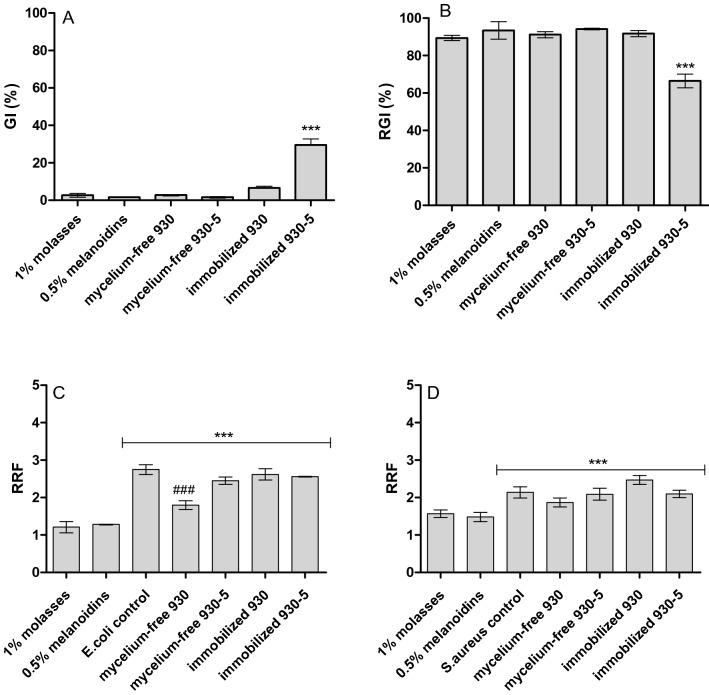
Germination index (**a**) and root growth inhibition (**b**) and resazurine reduction factors (C,D) of control solutions of 1% molasses and 0.5% melanoids and decolorized post-culture liquids of *B. adusta* CCBAS 930 from mycelium-free and immobilized cultures of *B. adusta* CCBAS 930 and 930-5 mutant; The significant difference was calculated using one-way ANOVA and post-Tukey test., ***p < 0.001 versus the control 1% molasses and melanoids medium, ### p < 0.001 versus the control growth of *E. coli* ATCC 25922 and *S. aureus* ATCC 29737; *GI* germination index, *RGI* root growth inhibition, *RRF* resazurine reduction factors

The results showed that the non-inoculated medium containing 1% molasses and 0.5% synthetic melanoid solutions inhibited seed germination (GI = 3.80–1.72%) and growth of roots (I = 89.41–95.43%) of *Lepidum sativum* L. (Fig. 8a, b). The decolorized post-culture liquids from the 21-day mycelium-free cultures of *B. adusta* CCBAS 930 and its mutant 930-5 were characterized by a similar level of inhibition of seed germination and root growth in *Lepidum sativum* L. as that in the non-inoculated control medium with 1% molasses and 0.5% melanoidins. The lowest phytotoxic effects was exhibited by decolorized post-culture liquids obtained after 7-day immobilized cultures of *B. adusta* CCBAS 930 and its 930-5 mutant (Fig. 8a, b). The tested strains had a higher germination index compared to the control 1% molasses and 0.5% melanoidins solution. The highest germination index and the lowest inhibition of root growth, i.e. GI = 86.31% and I = 62.23%, respectively, was recorded for *L. sativum* seeds in the presence of post-culture fluids obtained from the immobilized cultures of mutant 930-5 (Fig. 8a, b).

The biotoxicity assay with the use of Gram-positive *Staphylococcus aureus* ATCC 29737 and Gram-negative *Eschericha coli* ATCC 25,922 showed a lower value of the resasurine reduction factor (RRF = 1.21–1.52) in the initial media with the addition of 1% molasses and 0.5% melanoidin than in the control cultures of *Staphylococcus aureus* ATCC 29737 (RRF = 2.14) and *Eschericha coli* ATCC 25922 (RRF = 2.75) (Fig. 8c, d). This assay detecting the metabolic activity of bacterial cells demonstrated a similar RRF value for the supernatants from the mycelium-free cultures of *B. adusta* CCBAS 930 and its mutant 930-5 to that for the control bacteria, which suggested absence of biotoxicity of the supernatants (Fig. 7c, d).

### Changes in medium pH

In the first week of the stationary mycelium-free cultures (baseline pH 6.65), a decrease in the medium pH value was observed for all the strain cultures. The lowest decline was recorded for strain 930-5 (by 0.6 units). The pH value increased at the end of the third week of the cultivation of this strain. In the case of the other strains, the pH value of the medium increased gradually throughout the culture period. The pH in the immobilized cultures of the parental *B. adusta* CCBAS 930 and mutant 930-5 strains declined to a value of 5.63–5.85.

### Characterization of the growth of *B. adusta* CCBAS 930 and its mutants in molasses-supplemented and mycelium-free liquid media

Macroscopic observations of the mycelial growth in the molasses-containing liquid cultures revealed that all fungi developed inside the medium during the 1st week. The aerial mycelium appeared the earliest in the cultures of mutant 930-5, i.e. at the end of the 1st week (day 6), and covered 50% of the medium surface after 9 days. It persisted until the end of the experiment and was associated with decolorization. The other strains produced negligible amounts of aerial mycelium, which was visible only after 12 days of culture. The highest biomass values at the end of the culture (30 days) were achieved by mutants 930-5 and 930-14 (Table 3S).

## Discussion

The present study has shown that the ligninolytic anamorphic soil white-rot fungus *B. adusta* CCBAS930 strain capable of decolorization and decomposition of brown-colored humic acids and alkaline lignin derivative (Korniłłowicz-Kowalska et al. 2008) is able to decolorize and biodegrade melanoidins generated in the production of beet sugar and present in beet molasses. The ability of white-rot fungi to remove color from crude molasses and molasses spent wash, i.e. a waste product of the distillation of ethanol from molasses, was detected by Kim and Shoda (1998) in their investigations of *Geotrichum candidum* Dec1, which was reclassified as *Bjerkandera adusta* Dec1 after taxonomic verification and *Phanerochaete chrysosporium* (Sugano 2009; Yoshida et al. 2011; Vahabzadeh and Mehranian 2004; Thakker et al. 2006; Singh et al. 2007). Kim and Shoda (1998) demonstrated that strain Dec1 growing for 12 days in shaken cultures on medium containing 4% of molasses removed 87% of the color. The *B. adusta* CCBAS 930 strain investigated in the present study brightened the medium over the entire surface of the plate after 6 days of growth in agar medium containing 1% or 2% molasses. In turn, the medium supplemented with 1% molasses in the stationary liquid cultures was brightened only after 18 days by mutant 930-5, which removed over 30% of the color. The color loss in the parental strain cultures reached only 5%. Inhibition of molasses decolorization in stationary culture conditions by mycelium-free *Ph. chrysosporium* was reported by Thakkar et al. (2006). As shown in our previous studies, the decolorizing abilities of *B. adusta* CCBAS 930 (daunomycin, anthraquinone dyes, post-industrial lignin) are associated with the induction of peroxidase, i.e. an enzyme of the fungal secondary metabolism, which physiologically corresponds to the transition of the fungus to the differentiation stage (idiophase) associated with the emergence of sporulating aerial mycelium (Korniłłowicz-Kowalska et al. 2006a, b, 2008; Korniłłowicz-Kowalska and Rybczyńska 2010, 2014). This effect was pronounced in the mutant cultures, while vegetative growth (trophophase) was observed in the parental strain cultures. The more intensive mycelial growth observed in this study, including the aerial mycelium of mutant 930-5, compared with the parental strain, was also accompanied by more instantaneous utilization of reducing sugars and, as can be presumed, nitrogen required for mycelium formation. This induced faster transition to the idiophase stage and the related increase in peroxidase activity and molasses decolorization. This suggestion is supported by results reported by Kim and Shoda (1998) showing that strain *G. candidum* Dec1 decolorized molasses more efficiently in the presence of a lower dose of ammonium tartrate and that, at higher ammonium tartrate doses, the fungus utilized all sugar for mycelial growth after only 3–4 days with no decolorizing activity.

It was found that the decolorization of 1% molasses containing 0.5% melanoidins by mutant 930-5 in the mycelium-free cultures was accompanied by a 40% reduction in the level of melanoidins after a month of cultivation with no clear changes in the content of these substances in the parental strain cultures (data not shown). Nevertheless, the parental strain did not lose its ability to remove phenolic compounds. Although its ability was lower than that of mutant 930-5 (55% and 73% of reduction of the initial concentration after 18 days of culture, respectively), the parental strain exhibited higher efficiency of utilization of phenolic compounds in comparison with the other mutants (930-14; 930-20). A similar degree (74%) of reduction of the level of phenolic compounds by a microfungi *Penicillium decumbens* in beet molasses and molasses spent wash, i.e. wastewater from untreated molasses-based alcohol distilleries, was reported by Jimenez et al. (2003). Kim and Shoda (1998) demonstrated the presence of substances with a wide range of molecular weight in crude molasses. They reported that molasses decolorization by *G. candidum* Dec 1 involved decolorization of a fraction with higher molecular weight. Additionally, molasses contains a light orange low-molecular weight fraction, which is also transformed by strain Dec 1 (Kim and Shoda 1998). Gentisic acid, gallic acid, p-coumaric acid, quercetin etc. (Borja et al. 1993) as well as caramel and melanins (Satyawali and Balakrishan 2008) are small-molecule phenols contained in molasses. Given these data, we believe that strain *B. adusta* CCBAS 930 in the vegetative stage in the mycelium-free cultures may have utilized low-molecular phenols contained in molasses without utilization of the high-molecular fraction of melanoidins, which is responsible for the dark color and requires transition to the idiophase with the production of aerial mycelium.

The ability of such wood-rot fungi as *Phanerochaete chrysosporium*, *Trametes versicolor*, *Phlebia radiata*, or *Bjerkandera adusta* to remove color associated with the presence of lignin and many of its structurally related compounds (dyes with an aromatic structure, anthraquinone dyes, anti-cyclic antibiotics, humic acids, etc.) is determined by the fungal production of these substrate-unspecific oxidoreductive enzymes whose activity requires hydrogen peroxide produced with involvement of glucose oxidase or oxygen, i.e. peroxidase and laccase, respectively (Robinson et al. 2001; Moreira et al. 2007; Jarosz-Wilkołazka et al. 2002; Korniłłowicz-Kowalska and Rybczyńska 2012, 2014; Korniłłowicz-Kowalska et al. 2008). The present study demonstrated that strain *B. adusta* CCBAS 930 and its mutants growing in the presence of 1% molasses and 0.5% melanoids exhibited four different peroxidase activities: horseradish peroxidase-like HRP-like, manganese-dependent MnP, lignin LiP, and versatile VP peroxidases. In turn, they did not have laccase activity. No laccase activity was detected in *Bjerkandera* sp. Y-HHM2 cultures containing post-harvest sugarcane and molasses (Maza et al. 2015). Biosynthesis of MnP, LiP, and VP peroxidases by other *Bjerkandera* sp. and *B. adusta* strains were reported by Camarero et al. 1999, Moreira et al. 2006, 2007; Heinfling et al. 1998, and Master and Field 1998. Our study indicated that similar to *B. adusta* Dec 1 (previously referred to as *Geotrichum candidum* Dec 1), *B. adusta* CCBAS 930 was found to decolorize and decompose melanoids present in molasses with the involvement of dye-decolorizing peroxidase (Kim and Shoda 1998; Lee et al. 2000). Kim and Shoda (1998) reported that this process had a similar efficiency in mycelium-free (shaken) cultures and cultures of *B. adusta* Dec 1 cells immobilized on polyurethane foam. The present study showed that while the peroxidase activities in the mycelium-free cultures of mutant 930-5 were significant, with a maximum of HRP-like activity after 15 days of culture (0.7 U/mg protein and 2.81–3.22 U/mg protein in the case of VP), the activities in the parental strain and the other mutants were negligible. We suggested that this effect may have been caused by the difficulty in achievement of rapid transition from the vegetative stage to the mycelium differentiation stage (synthesis of sporulating aerial mycelium). The decolorization carried out with the use of the immobilized sporulating aerial mycelium of the parental strain (Na-alginate) confirmed this thesis. It was demonstrated that, in the immobilized cultures, strain CCBAS 930 contributed to 44.16% discoloration of a 1% molasses solution containing 0.5% melanoidins after 7 days, which was associated with strong stimulation of peroxidase activity, with the highest level of HRP-like activity (a 340-fold increase in comparison with the mycelium-free culture). An even stronger effect was observed in the case of mutant 930-5, which decolorized 30% of the 1% molasses solution after 3 days in immobilized cultures, and the maximum HRP-like peroxidase activity was over 640-fold higher than that of the parental strain in an analogous setup. After 7 days, the decolorization efficiency of 1% molasses in the immobilized cultures of this strain was 74.32%. Similar efficiency of decolorization of molasses spent wash by immobilized white-rot fungus *Flavodon flavus* was reported by Raghukumar et al. (2004). The authors showed that the polyurethane foam-immobilized fungus decolorized 60% to 73% of 10% brown-colored molasses spent wash after 5 and 7 days, respectively. Similarly, Fahy et al. (1997) obtained 60% decolorization of 6.25% diluted molasses spent wash in 8-day culture of *Phanerochaete chrysosporium* immobilized in alginate beads. Chavan et al. (2013) showed that immobilized mycelium of *Aspergillus oryzae* removed 75.71% of color in melanoidin-containing distillery spent wash effluents after 25 days. The decolorization of the distillery spent wash was also correlated with the MnP peroxidase activity and reduction of the phenolic content. Our previous research on the decolorization of various natural and synthetic anthraquinone derivatives by strain *B. adusta* CCBAS 930 (Korniłłowicz-Kowalska et al. 2006a, b, 2008; Korniłłowicz-Kowalska and Rybczyńska 2010, 2012, 2014) showed that the biodegradation of these aromatic color compounds with involvement of HRP-like peroxidase was accompanied by a decrease in the level of free radicals as well as hydroxy- and methoxyphenols. The present results confirm the key role of *B. adusta* CCBAS 930 HRP-like peroxidase in the decolorization and biodegradation of molasses melanoidins. The HRP-like activity in the immobilized cultures of *B. adusta* 930 and its mutant *B. adusta* 930-5 was 300 and 640-fold higher than in the mycelium-free cultures, respectively. This enzyme exhibited the highest potency of all the detected peroxidase activities in the immobilized cultures of the parental strain and mutant 930-5, which coincided with the decolorization of molasses, reduction of the levels of phenolic compounds and free radicals, and biodegradation of molasses. Our previously study showed higher decolorization efficient and activity of HRP-like peroxidases in immobilized cultures of *B. adusta* CCBAS 930 with 0.01% of anthraquinone dyes (Rybczyńska-Tkaczyk and Korniłłowicz-Kowalska 2020). A 50% decline in the content of phenolic compounds during decolorization of molasses spent wash by *Flavodon flavus* was reported by Raghukumar and Rivankar (2001). Our investigations indicate that the level of phenols in the immobilized cultures during decolorization of 1% molasses containing 0.5% melanoidins by strains *B. adusta* CCBAS 930 and 930-5 decreased and reached 65% after 7 days in the mutant cultures. The initial increase in the level of free radicals noted in the cultures of these strains was followed by an over 60–66% decrease. Similar free radical transformations and HRP-like peroxidase activity were demonstrated by Malarczyk et al. (1997) in a study of biodegradation of lignin-rich waste by strain *Geotrichum-like* R59 (currently *B. adusta* CCBAS 930) and by Korniłłowicz-Kowalska et al. (2006a, b) in experiments on the biodegradation of daunomycin. As suggested by Leonowicz et al. (2001), the process of lignin biodegradation by ligninolytic enzymes, i.e. laccase or peroxidases, is determined by the removal of free radicals from the environment, which are formed during the cleavage of phenolic and methoxyphenol groups in the process of depolymerization of this compound. Superoxide dismutase (SOD), whose increased activity in the *B. adusta* CCBAS 930 (= *Geotrichum-like* R59) cultures containing post-industrial lignin was correlated with an increase in peroxidase activity and a simultaneous decrease in phenolic compounds and free radicals, is involved in the removal of free radicals (Malarczyk et al. 1997). Therefore, the reduction of the level of free radicals and phenolic compounds accompanied by the increase in HRP-like peroxidase activity in the *B. adusta* CCBAS 930 and mutant 930-5 cultures during the decolorization of molasses melanoidins indicates a similar mechanism of biodegradation of these polymers to that of lignin and structurally related compounds such as humic acids and daunomycin (Belcarz et al. 2005; Korniłłowicz-Kowalska et al. 2006a, b; Malarczyk et al. 1997).

In an experiment with *Chlorella*, Chavan et al. (2013) demonstrated that biotreatment of melanoidin distillery spent wash effluent with free and immobilized *A. oryzae* eliminated the toxic effect caused by these compounds. Using free and immobilized mycelia of *Flavodon flavus* as a biological agent for decolorization of molasses spent wash (MSW), Raghukumar and Rivonkar (2001) and Raghukumar et al. (2004) achieved detoxification of MSW. The process of enzymatic decolorization and degradation of molasses melanoidins by the mycelium-free and immobilized mycelium of *B. adusta* CCBAS 930 and mutant 930-5 also resulted in a reduction of the toxicity of the decolorized post-culture liquids of these fungi measured in phyto- and biotoxicity assays (Gram + and Gram– bacteria). The detoxification of post-culture liquids accompanying the melanoidin biodegradation process indicates a potential use of extracellular HRP-like peroxidase from strain *B. adusta* CCBAS 930 and its mutant 930-5 for treatment of molasses as an industrial raw material. In turn, the idiophasic mycelium of both strains, e.g. in the immobilized form, can be used in management and detoxification of the waste biomass, i.e. molasses spent wash.

## Electronic supplementary material

Below is the link to the electronic supplementary material.

Supplementary file1 (DOC 40 kb)

Supplementary file2 (DOCX 13 kb)

Supplementary file3 (DOCX 16 kb)
