# Supplementary material for: Decolorization and biodegradation of melanoidin contained in beet molasses by an anamorphic strain of Bjerkandera adusta CCBAS930 and its mutants
Source: World J Microbiol Biotechnol. 2020 Dec 22;37(1):1. doi: 10.1007/s11274-020-02944-w (PMC7752745; doi:10.1007/s11274-020-02944-w)
Supplement: Supplementary file 1 — Supplementary file1 (DOC 40 kb) [file 11274_2020_2944_MOESM1_ESM.doc]

Table 1S. Chemical characterization of molasses.

| mineral compounds  (µg/g) | Na | K | Ca | Mg | Zn | Cu | Fe | Mn |
| --- | --- | --- | --- | --- | --- | --- | --- | --- |
|  | 3464.79 | 34658.44 | 1083.50 | 24.70 | 57.08 | 1.18 | 54.89 | 25.36 |
| protein | 13.76 | | | | | | | |
| nitrogen (%) | 2.20 | | | | | | | |
| reducing sugar  (mg/g) | 3.50 | | | | | | | |
| melanoids  (mg/g) | 50.00 | | | | | | | |
| amino acids (gAA/g of sample) | | | | | | | | |
| alanine | 2.6 | | | | | | | |
| arginine | 0.5 | | | | | | | |
| asparagine | 5.5 | | | | | | | |
| cysteine | 0.1 | | | | | | | |
| phenylalanine | 0.5 | | | | | | | |
| glycine | 2.3 | | | | | | | |
| glutamine | 49.5 | | | | | | | |
| histidine | 0.7 | | | | | | | |
| isoleucine | 2.9 | | | | | | | |
| leucine | 3.1 | | | | | | | |
| lysine | 0.5 | | | | | | | |
| methionine | 0.9 | | | | | | | |
| proline | 1.1 | | | | | | | |
| serine | 1.8 | | | | | | | |
| tryptophan | 0.8 | | | | | | | |
| tyrosine | 8.9 | | | | | | | |
| valine | 1.8 | | | | | | | |
| total | 83.3 | | | | | | | |
