# Supplementary material for: Decolorization and biodegradation of melanoidin contained in beet molasses by an anamorphic strain of Bjerkandera adusta CCBAS930 and its mutants
Source: World J Microbiol Biotechnol. 2020 Dec 22;37(1):1. doi: 10.1007/s11274-020-02944-w (PMC7752745; doi:10.1007/s11274-020-02944-w)
Supplement: Supplementary file 2 — Supplementary file2 (DOCX 13 kb) [file 11274_2020_2944_MOESM2_ESM.docx]

Tables 2S. Decolorization of molasses in solid Parka and Robinson medium by strain of
*B. adusta* CCBAS 930 and its mutants; *- diameter of growth in mm; ** - diameter of decolorization zone in mm (diameter of Petri dish - 90 mm)

| strains | molasses concentrations (%) | | | | | | | | | |
| --- | --- | --- | --- | --- | --- | --- | --- | --- | --- | --- |
|  | 1 | | | | | 2 | | | | |
|  | days of cultures | | | | | | | | | |
|  | 3 | 4 | 5 | 6 | 7 | 3 | 4 | 5 | 6 | 7 |
| 930  (parental) | 54*/0** | 80/40 | 90/50 | 90/90 | 90/90 | 48/0 | 65/45 | 80/80 | 90/90 | 90/90 |
| 930-2 | 42/0 | 57/0 | 75/0 | 90/0 | 90/20 | 32/0 | 47.5/0 | 60/0 | 90/0 | 90/0 |
| 930-5 | 19.5/0 | 50/0 | 80/20 | 90/90 | 90/90 | 30/0 | 30/0 | 32/0 | 57.5/10 | 90/20 |
| 930-14 | 32/0 | 40/0 | 57.5/0 | 80/10 | 90/50 | 20/0 | 22.5/0 | 57.5/0 | 62.5/0 | 90/0 |
| 930-20 | 30.5/0 | 42.5/0 | 60/0 | 90/0 | 90/10 | 28/0 | 40/0 | 50/0 | 80/0 | 90/0 |
