# Supplementary material for: Decolorization and biodegradation of melanoidin contained in beet molasses by an anamorphic strain of Bjerkandera adusta CCBAS930 and its mutants
Source: World J Microbiol Biotechnol. 2020 Dec 22;37(1):1. doi: 10.1007/s11274-020-02944-w (PMC7752745; doi:10.1007/s11274-020-02944-w)
Supplement: Supplementary file 3 — Supplementary file3 (DOCX 16 kb) [file 11274_2020_2944_MOESM3_ESM.docx]

Table 3S. Characteristics of the growth of parental strain of *B. adusta* CCBAS 930 and its mutants on molasses liquid media

| strains | days of cultures | | | | | | | | | |
| --- | --- | --- | --- | --- | --- | --- | --- | --- | --- | --- |
|  | 3 | 6 | 9 | 12 | 15 | 18 | 21 | 24 | 27 | 30 |
| 930  (parental) | **+ */ -**** | **++ / -** | **+++ / -** | **+++ / +** | **+++ / +** | **+++ / +** | **+++ / +** | **+++ / +** | **+++ / +** | **+++ / +** |
| 930-2 | **+ / -** | **++ / -** | **+++ / -** | **+++ / +** | **+++ / +** | **+++ / +** | **+++ / +** | **+++ / +** | **+++ / +** | **+++ / +** |
| 930-5 | **++ / -** | **+++ / +** | **+++ / ++** | **+++ / ++** | **+++ / ++** | **+++ / ++** | **+++ / ++** | **+++ / ++** | **+++ / ++** | **+++ / ++** |
| 930-14 | **+ / -** | **+++ / -** | **+++ / +** | **+++ / ++** | **+++ / ++** | **+++ / +** | **+++ / +** | **+++ / +** | **+++ / +** | **+++ / +** |
| 930-20 | **+ / -** | **++ / -** | **+++ / -** | **+++ / -** | **+++ / +** | **+++ / +** | **+++ / +** | **+++ / +** | **+++ / +** | **+++ / +** |

* - submerged mycelium, ** - aerial mycelium, (-) lack of growth; (+) weak growth of mycelium (20%), (++) moderate growth of mycelium (50%), (+++) strong growth of mycelium 80-100%, weak decolorization of molasses (10%), moderate decolorization of molasses 30-40%
